# Effect of Nd:YAG Laser Irradiation on the Growth of Oral Biofilm

**DOI:** 10.3390/microorganisms12112231

**Published:** 2024-11-04

**Authors:** Zuzanna Grzech-Leśniak, Jagoda Szwach, Martyna Lelonkiewicz, Krzysztof Migas, Jakub Pyrkosz, Maciej Szwajkowski, Patrycja Kosidło, Magdalena Pajączkowska, Rafał Wiench, Jacek Matys, Joanna Nowicka, Kinga Grzech-Leśniak

**Affiliations:** 1 Faculty of Medicine and Dentistry, Wroclaw Medical University, 50-367 Wroclaw, Poland; zuzanna.grzech-lesniak@student.umw.edu.pl; 2 Faculty of Medicine, Wroclaw Medical University, 50-367 Wroclaw, Poland; jagoda.szwach@student.umw.edu.pl (J.S.); martyna.lelonkiewicz@student.umw.edu.pl (M.L.); krzysztof.migas@student.umw.edu.pl (K.M.); jakub.pyrkosz@student.umw.edu.pl (J.P.); maciej.szwajkowski@student.umw.edu.pl (M.S.); patrycja.kosidlo@student.umw.edu.pl (P.K.); 3 Department of Microbiology, Faculty of Medicine, Wroclaw Medical University, 50-367 Wroclaw, Poland; magdalena.pajaczkowska@umw.edu.pl (M.P.); joanna.nowicka@umw.edu.pl (J.N.); 4 Department of Periodontal Diseases and Oral Mucosa Diseases, Faculty of Medical Sciences in Zabrze, Medical University of Silesia, 40-055 Katowice, Poland; rwiench@sum.edu.pl; 5 Laser Laboratory, Department of Dental Surgery, Faculty of Dentistry, Wroclaw Medical University, 50-425 Wroclaw, Poland; jacek.matys@umw.edu.pl; 6 Department of Periodontics, School of Dentistry, Virginia Commonwealth University VCU, Richmond, VA 23298, USA

**Keywords:** *Candida* spp., neodymium laser, oral biofilm, *Streptococcus mutans*

## Abstract

Background: Oral microbiota comprises a wide variety of microorganisms. The purpose of this study was to evaluate the effects of Nd:YAG laser with a 1064 nm wavelength on the in vitro growth of *Candida albicans*, *Candida glabrata*, and *Streptococcus mutans* clinical strains, as well as their biofilm. The study also aimed to determine whether the parameters recommended for photobiomodulation (PBM) therapy, typically used for tissue wound healing, have any additional antibacterial or antifungal effects. Material and Methods: Single- and dual-species planktonic cell solution and biofilm cultures of *Streptococcus mutans*, *Candida albicans*, and *Candida glabrata* were irradiated using an Nd:YAG laser (LightWalker; Fotona; Slovenia) with a flat-top Genova handpiece. Two test groups were evaluated: Group 1 (G-T1) exposed to low power associated parameters (irradiance 0.5 W/cm^2^) and Group 2 (G-T2) with higher laser parameters (irradiance 1.75 W/cm^2^). Group 3 (control) was not exposed to any irradiation. The lasers’ effect was assessed both immediately after irradiation (DLI; Direct Laser Irradiation) and 24 h post-irradiation (24hLI) of the planktonic suspension using a quantitative method (colony-forming units per 1 mL of suspension; CFU/mL), and the results were compared with the control group, in which no laser was applied. The impact of laser irradiation on biofilm biomass was assessed immediately after laser irradiation using the crystal violet method. Results: Nd:YAG laser irradiation with photobiomodulation setting demonstrated an antimicrobial effect with the greatest immediate reduction observed in *S. mutans*, achieving up to 85.4% reduction at the T2 settings. However, the laser’s effectiveness diminished after 24 h. In single biofilm cultures, the highest reductions were noted for *C. albicans* and *S. mutans* at the T2 settings, with *C. albicans* achieving a 92.6 ± 3.3% reduction and *S. mutans* reaching a 94.3 ± 5.0% reduction. Overall, the T2 settings resulted in greater microbial reductions compared to T1, particularly in biofilm cultures, although the effectiveness varied depending on the microorganism and culture type. Laser irradiation, assessed immediately after using the crystal violet method, showed the strongest biofilm reduction for *Streptococcus mutans* in the T2 settings for both single-species and dual-species biofilms, with higher reductions observed in all the microbial samples at the T2 laser parameters (*p* < 0.05) Conclusion: The Nd:YAG laser using standard parameters typically applied for wound healing and analgesic effects significantly reduced the number of *Candida albicans*; *Candida glabrata*; and *Streptococcus mutans* strains.

## 1. Introduction

The oral cavity is a complex ecosystem, and any disruption to the microbial balance can pose challenges to both local and systemic health. The primary dental diseases resulting from microbial dysbiosis include caries, gingivitis, and periodontitis. *Candida* spp. and *Streptococcus mutans* are capable of forming dual-species biofilms, especially in conditions of oral dysbiosis, where the equilibrium of the oral microbiome is disrupted [1,2]. *Streptococcus* species are the predominant genus found in saliva and oral soft tissues, with *S. mutans* playing a key role in the development of dental caries [3]. *Candida albicans* and *Candida glabrata* are potentially pathogenic yeasts that predispose individuals to oral candidiasis, particularly in vulnerable patients such as those who are immunosuppressed, have systemic diseases like diabetes, or use steroid inhalers [4,5]. Therefore, it is essential to explore safe and advanced treatment methods targeting these species as alternatives to conventional antibiotic or antifungal therapies, which are associated with a range of side effects.

Microorganisms in the oral cavity are often organized into biofilms—communities of bacteria or fungi attached to surfaces and protected by an extracellular matrix. They can also exist in planktonic forms, allowing them to freely float in saliva or other fluids. Artificial research biofilms composed of multiple species more closely resemble those found naturally in the oral cavity, making them essential to include in studies. *Streptococcus mutans* and *Candida albicans* interact within biofilm structures, enhancing cariogenic effects and contributing to peri-implantitis [6,7]. Biofilm structures are highly resistant to antibiotics highlighting the need for new methods and guidelines to effectively eliminate biofilms [8]. One of the main challenges in developing new anti-biofilm agents is their potential systemic toxicity, which must be carefully considered [6]. Therefore, exploring treatment options with fewer contraindications and minimal side effects, such as laser therapies, is crucial.

Laser therapies are utilized across various fields in dentistry [9,10,11,12,13,14,15,16,17,18,19]. The antimicrobial mechanism of lasers is attributed to photothermal and photochemical reactions. Nd:YAG lasers have been employed in the treatment of periodontitis due to their proven efficacy in reducing periodontal pathogens [11,12,16,19,20,21,22,23]. According to some studies, Nd:YAG laser light reduces the adhesion of *S. mutans*, potentially interfering with its biofilm formation [24]. However, research on the effects of Nd:YAG lasers on *Candida albicans* biofilms remains inconsistent. For instance, Kasić et al. reported that the Nd:YAG laser does not exhibit activity against *Candida albicans* biofilm [9,25,26,27], while other researchers have demonstrated its antifungal properties and ability to disrupt the biofilm structures [24,25,26,27,28]. These discrepancies among studies, along with a shortage of recent research, highlight the need for further investigation into the effectiveness of laser treatments.

The purpose of this study was to evaluate the effects of a 1064 nm Nd:YAG laser on the in vitro growth of clinical strains, including *Candida albicans* (C.a.), *Candida glabrata* (C.g.), and *Streptococcus mutans* (S.m.), in both single- and dual-species planktonic and biofilm formations to explore new treatment options against these species. It is important to emphasize that the parameters used in this research are currently employed in clinical protocols for their analgesic effects and are specifically designed to enhance wound healing through photobiomodulation therapy.

## 2. Materials and Methods

The study was approved by the Bioethics Committee of the Medical University of Wroclaw (KB- 429/2024).

### 2.1. Sample Preparation

The study was conducted on clinical strains of the microorganisms, part of the strain collection at the Department of Microbiology, Wroclaw Medical University, Poland. The strains were obtained from swabs taken from the dorsal part of the tongue of patients diagnosed with oral candidiasis: *C. albicans* (C.a.) and *C. glabrata* (C.g.), and from patients with carious lesions: *S. mutans* (S.m.).

The microorganisms were stored in trypticase soy broth (Biomaxima, Lublin, Poland) in temp −80 °C, with each strain frozen in several replicates.

Before each experimental cycle, the strains were cultured under conditions appropriate for each microorganism:

- *Candida* spp. on Sabouraud Dextrose Broth agar (Biomaxima, Lublin, Poland) at 37 °C for 48 h in aerobic conditions.
- *S. mutans* on Brain Heart Infusion (BHI) agar (Biomaxima, Lublin, Poland) at 37 °C for 48 h under elevated CO_2_ levels.

To enhance the differentiation of the results, two types of samples were prepared:

1. Planktonic Solutions of Cells—Appropriate amounts of microorganisms’ suspension (0.5 McFarland standard) were prepared: 100 µL of the suspension was combined with 900 µL of BHI broth with 5% sucrose (Biomaxima, Lublin, Poland) for *C. albicans* (C.a.), *C. glabrata* (C.g.), and *S. mutans* (S.m.) (see Table 1); 100 µL of each microorganism and 800 µL of BHI broth for the following combinations: *C. albicans + S. mutans*, *C. glabrata* + *S. mutans*, and *C. albicans* + *C. glabrata* (see Table 2). All the samples were treated with laser light while contained in a dark Eppendorf tube. Dark Eppendorf tubes were used to ensure that no external light could affect the material inside the tube. Additionally, the dark color of the tubes helps block some of the scattered PBM light, preventing it from influencing other Eppendorf tubes that were not irradiated.

2. Biofilms—Experiments were conducted using two biofilm models: single-species and dual-species. The biofilm formation method involved the use of flat-bottom 96-well polystyrene plates (FL Medical, Equimed, Krakow, Poland). Aliquots of 100 µL of microorganism suspension, with a density of 0.5 according to the McFarland standard, were added to each well of the titration plate, along with 150 µL of liquid BHI broth with 5% sucrose (Biomaxima, Lublin, Poland), resulting in a final volume of 250 µL for single-species suspensions (Table 3).

For two-species suspensions, 100 µL of the first microorganism suspension and 100 µL of the second microorganism (both with a density of 0.5 on the MacFarland standard) were added to the wells of the titration plate. This was supplemented with 50 µL of liquid BHI broth containing 5% sucrose (Biomaxima, Lublin, Poland), resulting in a final volume of 250 µL (see Table 4).

All the plates were incubated at 37 °C for 24 h under aerobic conditions for yeast or in elevated 5% CO_2_ levels for *Streptococcus mutans*. The resulting biofilm was formed on the bottom and walls of the wells.

### 2.2. Laser Application

**Figure 1:**
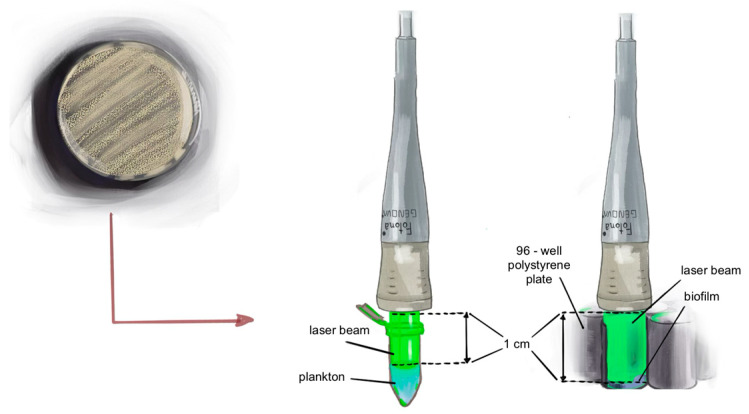
Diagram of methodology of laser irradiation of planktonic solutions of cells and biofilm.

This study utilized a near-infrared neodymium-doped yttrium aluminum garnet, Nd:YAG laser (LightWalker, Ljubljana, Fotona, Slovenia) operating at a wavelength of 1064 nm using a flat-top handpiece (Genova, LightWalker, Ljubljana, Fotona, Slovenia). The handpiece was maintained at a constant distance of 1 cm from the surface of the suspension or the edge of the wall containing the biofilm (Figure 1).

The investigation comprised two sets of parameters across two experimental groups (G-T1 and G-T2):

- Group 1 (G-T1): Power 0.48 W, irradiance 0.5 W/cm^2^, fluence 50 mJ/cm^2^, frequency 10 Hz, spot diameter 11 mm, spot area 0.95 cm^2^, total dose 29 J, irradiation time 60 s, and a Micro Short Pulse (MSP) of 150 µs. These parameters are recommended by the manufacturer for wound healing.
- Group 2 (G-T2): Power 1.62 W, irradiance 1.75 W/cm^2^, fluence 58 mJ/cm^2^, frequency 30 Hz, spot diameter 11 mm, spot area 0.95 cm^2^, total dose 100 J, and irradiation time 60 s, with MSP 150 µs. These parameters are recommended by the manufacturer for pain relief (analgesic effect).
- Group 3 (control): Non-irradiated samples.

### 2.3. Microorganism Quantification

#### 2.3.1. Reduction in CFU/mL Colony-Forming Units Under the Influence of Laser Effect Immediately After Application

Immediately following the laser irradiation of the dark Eppendorf tubes containing different single- or dual-species inoculum, 100 µL of the suspension was extracted using a pipette and diluted in geometric progression. From the final dilution, 0.1 µL of the suspension was plated onto solid BHI agar and incubated (37 °C for 24 h) under aerobic conditions for yeast or elevated CO_2_ conditions for *Streptococcus mutans*. The colonies that grew were counted, and the CFU/mL value was calculated. The preparation of the control inoculum followed the same procedure as the experimental samples but without irradiation.

#### 2.3.2. Reduction in CFU/mL Colony-Forming Units Under the Influence of Laser Effect 24 h After Application

Following the laser irradiation of the dark Eppendorf tubes containing various single or dual-species inoculum, the suspension underwent an incubation period at 37 °C for 24 h under aerobic conditions for yeast and elevated CO_2_ conditions for *Streptococcus mutans*. After incubation, 100 µL of the suspension was extracted using a pipette and diluted in geometric progression. From the final dilution, 0.1 µL of the suspension was plated onto a solid BHI agar and incubated again (37 °C for 24 h) under the same respective conditions. The colonies that developed were counted, and the CFU/mL values were calculated. The preparation of the control inoculum followed the same procedure as the experimental samples but without irradiation.

#### 2.3.3. Evaluation of the Laser Effect on the Eradication of Single- and Dual-Species Biofilm (Quantitative Method—Reduction in CFU/mL Values)

After preparing fresh single- and dual-species biofilms as described in Section 2.1, the wells were rinsed three times with 0.9% NaCl solution to remove the microbial cells that were not attached to the biofilm. The biofilm was then irradiated, and it was scraped off immediately after laser exposure using a sterile swab. The swab was subsequently agitated for 1 min in a 0.5% saponin solution (Sigma-Adrich, Saint Louis, MO, USA).

A total of 100 µL of the resulting suspension was quantitatively inoculated onto a solid substrate and incubated at 37 °C for 24 h under aerobic conditions for yeast or elevated CO_2_ conditions for *Streptococcus mutans*. The colonies that developed were counted, and the CFU/mL values were calculated.

The control group consisted of biofilms that did not receive laser irradiation.

#### 2.3.4. Evaluation of the Laser Effect on Biofilm Biomass: Crystal Violet Method

Following the preparation of fresh single- and dual-species biofilms as described in Section 2.1, the wells were rinsed three times with 0.9% NaCl solution to remove the microbial cells that were not attached to the biofilm. The biofilm was then subjected to laser irradiation. After the application of the laser, the biofilm was fixed by drying for 45 min at room temperature. Subsequently, 250 µL of 0.1% crystal violet solution (Chempur, Piekary Śląskie, Poland) was added to each well, and the plate was left at room temperature for 20 min. After removing the crystal violet, the wells were rinsed three times with distilled water and allowed to dry for an additional 20 min at room temperature. To quantify the biomass, 200 μL of 95% ethanol (Honeywell, Charlotte, NC, USA) was added to each well, and the absorbance was measured at λ = 540 nm (Biochrom Asys UVM 340 ELISA reader; Biochrom Ltd., Fullerton, CA, USA). The control group underwent the same procedure as the experimental samples but did not receive laser irradiation.

### 2.4. Statistical Analysis

The Kolmogorov–Smirnov test was conducted at a 95% confidence level to assess the normality of the data distribution. A multivariate analysis of variance (ANOVA) was employed to compare microorganism reduction after laser irradiation. All the statistical analyses were performed using the Statistica software (version 12, StatSoft, Krakow, Poland), with the significance level set at *p* < 0.05.

## 3. Results

### 3.1. Microorganism Reduction in Single-Species Planktonic Cultures

The greatest reduction in microorganism cell counts (CFU/ml) was observed immediately following the laser application for *S. mutans*, with reductions of 84.6 ± 7.6% and 85.4 ± 1.0% for the T1 and T2 laser settings, respectively. However, 24 h post-irradiation, the levels of microorganisms showed a slight, non-significant decrease (*p* > 0.05) for all the species at the T2 settings, except *S. mutans*, where the eradication effect of the Nd:YAG laser with the Genova handpiece at the T2 settings significantly diminished (*p* < 0.05). Furthermore, a significantly greater reduction in C. albicans was noted when using the Genova handpiece immediately after laser application at the T1 setting. Similarly, a greater reduction at the T1 setting was seen for *S. mutans* 24 h after laser application. Immediately following laser application, the reduction in *S. mutans* was significantly higher compared to *C. albicans* at both laser settings and compared to *C. glabrata* at the T1 setting (Table 5).

### 3.2. Microorganism Reduction in Single-Species Biofilm Cultures

The greatest reduction in single species in the biofilm was observed at the T2 settings for *C. albicans* (T1-68.4 ± 2.3%, T2-92.6 ± 3.3%) (*p* < 0.05) and *S. mutans* (T1-33.1 ± 5.9%, T2-94.3 ± 5.0%) (*p* < 0.05). Additionally, a significantly greater reduction in *C. albicans* levels compared to *S. mutans* in the T1 laser settings was noted directly after laser application (*p* < 0.05) (Table 6).

### 3.3. Microorganism Reduction in Two-Species Planktonic Cultures

The highest reduction in microorganism cell counts (CFU/mL) was observed immediately after laser application at the T2 settings for *C. albicans* (C.a.) and *S. mutans* (S.m.), with significant reductions of 84.1 ± 2.2% and 81.8 ± 3.4%, respectively (*p* < 0.05). Additionally, the reduction in *S. mutans* CFU/mL levels was significantly greater in the *C. glabrata* + *S. mutans* (C.g. + S.m.) mixture at the T2 settings, but notably lower in the *C. albicans* + *S. mutans* (C.a. + S.m.) mixture at the T1 settings immediately after laser application (*p* < 0.05). A decrease in the laser’s microbial reduction effect was observed 24 h after irradiation in almost all the samples (Table 7).

### 3.4. Microorganism Reduction in Two-Species Biofilm Cultures

In all the samples after laser irradiation at the T2 settings, a high reduction in microbial levels was observed, ranging from 96.4 ± 4.9% to 100 ± 0.0%. A significantly greater reduction in *C. glabrata (C.g.)* levels between the two settings was noted in the *C. albicans* + *C. glabrata* C.a. + C.g. mixture biofilm (*p* < 0.05). Furthermore, the analysis of single-species levels within dual-species biofilm indicated a significantly greater reduction for *C. albicans* compared to *C. glabrata* at the T1 settings (*p* < 0.05) (Table 8).

### 3.5. Microorganism Reduction in Single- and Two-Species Biofilm Cultures (Crystal Violet Method)

The impact of laser irradiation on biofilm biomass was assessed immediately after laser irradiation using the crystal violet method. The strongest reduction in single-species biofilms was observed for *Streptococcus mutans* in the T2 laser setting (Table 9), and for the dual-species biofilms (Table 10).

Reduction in the simple-species biofilm culture in all the microbial samples was higher for the T2 laser parameters. The reduction in *Streptococcus mutans* was greater and statistically significant (*p* < 0.05) compared to *Candida albicans* and *Candida glabrata*.

Reduction in the two-species biofilm culture in all the microbial samples was higher for the T2 laser parameters. Reduction in *Candida albicans* was higher in biofilms: C.a. + S.m. and C.a. + C.g. For *Candida glabrata* and *Candida albicans* biofilms, the reduction was statistically significantly greater than for *Streptococcus mutans* in biofilms (*p* < 0.05).

## 4. Discussion

Neodymium lasers have been widely used in various fields of medicine, including the clinical treatment of pain, infections, and the stimulation of wound healing processes [29,30,31]. This study aimed to evaluate the antimicrobial effect of the Nd:YAG laser against C. albicans (C.a.), C. glabrata (C.g.), and *S. mutans* (S.m.) in both planktonic and biofilm cultures using clinical parameters typically applied in photobiomodulation therapy. This study demonstrated that the Nd:YAG laser, equipped with the Genova handpiece, significantly reduced microorganism levels across various single- and dual-species planktonic and biofilm cultures. 

In single-species planktonic cultures, the most pronounced reductions in CFU/mL occurred immediately after laser application, particularly for *S. mutans*, where reductions reached up to 85.4 ± 1.0% at the T2 setting. Although most microorganisms exhibited a slight, non-significant decrease after irradiation, the effectiveness of the laser against *S. mutans* significantly diminished at the T2 setting (see Table 5). In single-species biofilm cultures, the largest reductions were observed directly after laser application, especially for C. albicans and *S. mutans*, with the greatest impact at the T2 settings (see Table 6). For two-species planktonic cultures, both C. albicans and *S. mutans* experienced significant reductions immediately after laser treatment, particularly at higher T2 settings (see Table 7). In dual-species biofilm cultures, microbial levels were reduced by 96.4 ± 4.9% to 100±0.0% at the higher T2 setting directly after laser irradiation, with C. albicans showing a significantly greater reduction compared to C. glabrata at T1 settings (see Table 8). 

However, to optimize the therapeutic potential of the Nd:YAG laser, ongoing research should explore a wide range of parameters and applicators to ensure uniform energy distribution across the irradiated surface.

*Candida albicans* is the primary etiological agent of oral candidiasis, though the role of non-*albicans* species as infection-causing agents is increasingly recognized. Colonization by multiple *Candida* species is more common with the use of prosthetic restorations, with *C. albicans* and *C. glabrata* being the most frequently isolated species in mixed infections [32]. In this study, we evaluated the activity of the Nd:YAG laser against the bacterium *Streptococcus mutans* and the yeast *C. albicans* and *C. glabrata*, assessing its effects on both planktonic forms and fungal biofilm biomass. Research by Ying Jao et al. shows that Candida species, which can develop resistance to pharmacotherapies over time, can be more effectively eradicated when combined with a low-power Nd:YAG laser [33]. Our analysis of single-species levels in dual-species biofilm indicated a significantly greater reduction for *Candida albicans* compared to *Candida glabrata* at a fluence of 50 mJ/cm^2^ (power 0.48 W, irradiance 0.5 W/cm^2^).

Furthermore, a study by Namour M. et al. demonstrated a statistically significant effect of the Q-Switch Nd:YAG laser, with parameters of energy density 0.597 J/cm^2^, 0.27 W, 2.4 mm spot diameter, and 10 Hz, in reducing the growth of multispecies biofilm on titanium plates. The laser-treated plate showed no significant difference compared to the test sample with laser exposure alone or the untreated plate [34]. Our study also examined the laser’s effect on the growth of co-infecting colonies: *C. albicans* + *S. mutans* (C.a. + S.m.), *C. glabrata* + *S. mutans* (C.g. + S.m.), and *C. albicans* + *C. glabrata* (C.a. + C.g.), in both planktonic and biomass colonies. Statistical analysis revealed that reductions in two-species colonies were influenced not only by the specific laser parameters but also by the mutual interactions between the combined microorganisms.

Baroni et al. demonstrated the effect of Q-switched Nd:YAG 1064 nm laser on the reduction in *C. albicans* colonies, achieving 63% reduction 24 h post-treatment with an energy density of 8 J/cm^2^ [9]. Similarly, Maden et al. exposed *C. glabrata* colonies to the Nd:YAG laser, reporting reductions of 78.06% (1.5 W), 96.13% (1.8 W), and 100% (2 W) after applying 15 pulses per sample [26]. In an in vitro study, Dubravko Risovic et al. indicated that the survival of *C. albicans* colonies depends strictly on the laser parameters used, with the most effective wavelength for eradication being UV-C 254 nm (Δ = 6.1 mJ/cm^2^, ET99.99 = 56 mJ/cm^2^) and the least efficient 406 nm (Δ = 11.4 J/cm^2^, ET99.99 = 104 J/cm^2^) [35].

Our study also demonstrated a statistically significant reduction effect of the Nd:YAG laser on both planktonic and biofilm cultures. The most effective results for planktonic cultures were observed with *C. glabrata* (G-T2, 24AI, 81.6 ± 0.3%), while for biofilm colonies, the highest reductions were seen with *C. albicans* (G-T2, 24AI, 92.6 ± 3.3%) and *C. glabrata* (G-T2, 24AI, 94.3 ± 5.0%). The outcomes in specific cases were significantly influenced by variables such as the handpiece used, the time elapsed after irradiation, and the two different parameter settings applied. These variations highlight the importance of selecting appropriate laser parameters to optimize therapeutic efficacy.

The present study yielded significant findings regarding the effects of Nd:YAG laser treatment on *Streptococcus mutans* under various conditions. Immediately after laser application, *S. mutans* exhibited substantial reductions, with CFU/mL decreases of 84.6 ± 7.6% at the T1 settings and 85.4 ± 1.0% at the T2 settings. However, 24 h post-irradiation, the effectiveness of the Nd:YAG laser at the T2 settings significantly declined (*p* < 0.05), leading to a rebound in *S. mutans* levels compared to the immediate post-treatment results. The lack of change in the *S. mutans* population under the T2 conditions 24 h after irradiation suggests that the initial antimicrobial effect of the Nd:YAG laser diminished over time. Although the laser effectively reduced bacterial levels immediately after application, the bacteria may have demonstrated resilience, possibly through biofilm formation or recovery mechanisms, allowing the population to stabilize after 24 h. This result indicates that while the laser can provide short-term bactericidal effects, it may not sustain long-term reductions in *S. mutans* at the applied T2 settings, highlighting the need for the optimization of the laser parameters or combination with other treatments for prolonged antimicrobial action. In single-species biofilm cultures, the T2 setting achieved a 94.3 ± 5.0% reduction in *S. mutans*, a notable improvement over the 33.1 ± 5.9% reduction observed at the T1 settings. Similarly, in two-species planktonic cultures, the T2 settings led to significant reductions, with an 81.8 ± 3.4% decrease in *S. mutans*. Despite these initial reductions, the long-term effectiveness of the laser in controlling *S. mutans* was less consistent, particularly in the T2 settings, indicating a diminished efficacy over time. Golob Deeb J et al. demonstrated that the combination of chlorhexidine (CHX) therapy with Nd:YAG laser treatment effectively slows the growth and pathogenicity of *S. mutans* [16]. In an in vitro study, Luciano Pereira Rosa showed that a 455 nm blue light-emitting diode (LED), when applied for varying durations, effectively reduces *Staphylococcus aureus* loads [36]. Alex M. Valm’s research highlights the importance of multifactorial interactions between microbial communities, the host immune system, and various environmental and host factors in microbial pathogenesis [37]. Additionally, Grzech-Leśniak et al. found that Nd:YAG laser light at a wavelength of 1064 nm induces the photoexcitation of endogenous microbial porphyrin molecules in *S. mutans* and *C. albicans*, resulting in oxidative damage via reactive oxygen species (ROS) generation [25].

Comparing the T1 (power 0.48 W, irradiance 0.5 W/cm^2^, and fluence 50 mJ/cm^2^) and T2 (power 1.62 W, irradiance 1.75 W/cm^2^, and fluence 58 mJ/cm^2^) laser settings across different microorganism cultures reveals distinct differences in efficacy. In single-species planktonic cultures, both settings exhibited similar initial reductions in *Streptococcus mutans* immediately after laser application, with T1 achieving a reduction of 84.6 ± 7.6% and T2 slightly higher at 85.4 ± 1.0%. However, 24 h post-irradiation, the effectiveness of the T2 setting significantly diminished for *S. mutans* (*p* < 0.05), while T1 maintained a more consistent reduction across all the microorganisms.

In single-species biofilm cultures, the T2 setting outperformed T1, particularly in reducing *Candida albicans* and *Streptococcus mutans* directly after laser irradiation, with T2 achieving reductions of 92.6 ± 3.3% and 94.3 ± 5.0%, respectively, compared to T1’s 68.4 ± 2.3% and 33.1 ± 5.9%. The T2 setting also demonstrated superior performance in two-species planktonic and biofilm cultures, especially in reducing *C. albicans* and *C. glabrata*, where T2 consistently achieved higher reductions. However, the T1 setting proved more effective in specific instances, such as in reducing *S. mutans* levels in certain dual-species mixtures immediately after application.

Overall, while T2 generally provided higher reductions, T1 offered more consistent results over time. Based on the results presented in this study, the application of Nd:YAG laser treatment in clinical settings shows promising potential for managing localized microbial infections, particularly in the oral cavity. Our findings indicate that laser irradiation significantly reduces microbial viability, especially in single-species and two-species biofilm cultures, with the most substantial reductions observed immediately following treatment. This efficacy suggests that Nd:YAG laser therapy could serve as an effective adjunct to conventional antimicrobial therapies, particularly for patients with periodontal diseases and candidiasis.

While this study provides valuable insights into the efficacy of Nd:YAG laser treatment for microbial reduction, several limitations must be acknowledged. First, the short-term observation period may not fully capture the long-term effects of laser treatment, particularly regarding how microorganism levels stabilize or rebound over extended durations. Additionally, the results are based on specific laser settings (T1 and T2) and handpiece used, which may not encompass the full range of possible treatment parameters or equipment variations that could influence outcomes. The study primarily focuses on planktonic and biofilm cultures under controlled laboratory conditions, which may not fully replicate the complexity of clinical environments, where factors such as biofilm maturation and host interactions play significant roles. Moreover, while substantial reductions were observed, the variability in microbial responses at different settings and time points underscores the necessity for further research to optimize laser parameters and understand the underlying mechanisms of microbial resistance or recovery. Furthermore, the reliance on specific microbial strains limits the generalizability of the findings to other potentially relevant species or mixed microbial communities. Variability in microbial responses to Nd:YAG laser irradiation may hinder the applicability of the findings across different species and clinical settings. Thus, further research is needed to optimize laser parameters for broader clinical applications. Finally, clinical trials are necessary to confirm the effectiveness of Nd:YAG laser treatment in vivo.

## 5. Conclusions

Based on the results of this study, the Nd:YAG laser demonstrated significant antimicrobial effects across various settings and microorganism types. The laser was particularly effective in reducing the levels of *Streptococcus mutans* and *Candida albicans*, both in single-species planktonic cultures and biofilm formations, especially at higher power settings (T2). However, the effectiveness of microbial reduction diminished over time, with a noticeable decrease in eradication efficiency observed 24 h post-irradiation, particularly for *S. mutans* at the T2 settings.

In dual-species biofilms, the laser’s efficacy varied depending on the specific combination of microorganisms, with a more substantial reduction observed in the *C. glabrata* levels within the *C. albicans* and C. glabrata mixture at higher power settings. These findings underscore the importance of optimizing laser parameters and treatment protocols to maximize antimicrobial effects, particularly in complex biofilm environments. Further clinical research is needed to investigate the short- and long-term efficacy and potential applications of Nd:YAG laser treatment in clinical settings.

## Figures and Tables

**Table 1:** Proportion of suspension of strains and medium for single species.

Single Species	Suspension *	Substrate **
*Candida albicans*	100 µL	900 µL
*Candida glabrata*	100 µL	900 µL
*Streptococcus mutans*	100 µL	900 µL

* suspension with a density of 0.5 on the McFarland standard; ** liquid medium BHI broth with 5% sucrose.

**Table 2:** Proportion of suspension of strains and substrate for two-species suspension.

Two-Species Suspension	Suspension *	Substrate **
*Candida albicans* *Streptococcus mutans*	200 µL	800 µL
*Candida glabrata* *Streptococcus mutans*	200 µL	800 µL
*Candida albicans* *Candida glabrata*	200 µL	800 µL

* 0.5 McFarland density suspension—100 µL of each microorganism; ** liquid medium BHI broth with 5% sucrose.

**Table 3:** Proportion of suspension of strains and substrate for single-species biofilm.

Single-Species Biofilm	Suspension *	Substrate **
*Candida albicans*	100 µL	150 µL
*Candida glabrata*	100 µL	150 µL
*Streptococcus mutans*	100 µL	150 µL

* suspension with a density of 0.5 on the McFarland standard; ** liquid medium BHI broth with 5% sucrose.

**Table 4:** Proportion of suspension of strains and medium for two-species biofilm.

Two-Species Biofilm	Suspension *	Substrate **
*Candida albicans* *Streptococcus mutans*	200 µL	50 µL
*Candida glabrata* *Streptococcus mutans*	200 µL	50 µL
*Candida albicans* *Candida glabrata*	200 µL	50 µL

* 0.5 McFarland density suspension—100 µL of each microorganism; **—liquid medium BHI broth with 5% sucrose.

**Table 5:** Percentage change (%) in the number of microorganism cells (CFU/mL) after laser application, compared to the control group (non-irradiated samples), for single-species planktonic cultures.

Handpiece	Time	Bacteria	Reduction in %CFU/mL Mean (SD)		*p*-Value T1 vs. T2	*p*-Value C.a. vs. C.g. vs. S.m.	*p*-Value DAI vs. 24AI
			T1	T2			
Genova	DAI	*C. albicans*	53.5 (4.9)	31.9 (4.2)	0.043 *	C.a. vs. C.g. T1 0.059, T2 0.238 C.g. vs. S.m. T1 0.014 *, T2 0.228 C.a. vs. S.m. T1 0.040 *, T2 0.003 *	*C. albicans* T1 0.123, T2 0.687 *C. glabrata * T1 0.066, T2 0.077 *S. mutans* T1 0.277, T2 0.000 *
		*C. glabrata*	38.9 (1.7)	58.5 (22.1)	0.339		
		*S. mutans*	84.6 (7.6)	85.4 (1.0)	0.897		
	24AI	*C. albicans*	43.2 (2.7)	52.9 (7.6)	0.232	C.a. vs. C.g. T1 0.814, T2 0.034 * C.g. vs. S.m. T1 0.917, T2 0.000 * C.a. vs. S.m. T1 0.763, T2 0.000 *	
		*C. glabrata*	49.1 (30.8)	81.6 (0.3)	0.273		
		*S. mutans*	46.3 (12.4)	0.0 (0.0)	0.034 *		

DAI—directly after irradiation; 24AI—24 h after irradiation; T1—laser settings (lower power); T2—laser settings (higher power); C.a.—Candida albicans; C.g.—Candida glabrata; S.m.—Strepto-coccus mutans; reduction in %CFU/mL—percentage reduction in the number of colony-forming units (CFU)/mL; SD—standard deviation; *—Statistical significance (*p* < 0.05).

**Table 6:** Percentage change (%) in the number of microbial cells (CFU/mL) after Nd:YAG laser application, compared to the control group (non-irradiated samples), for the C.g. + S.m., C.a. + S.m., and C.a. + C.g. biofilm mixtures.

Headpiece	Time	Bacteria	Reduction in %CFU/mL Mean (SD)		*p*-Value T1 vs. T2	*p*-Value C.a. vs. C.g. vs. S.m.
			T1	T2		
Genova	DAI	*C. albicans*	68.4 (2.3)	92.6 (3.3)	0.013 *	C.a. vs. C.g. T1 0.758, T2 0.734 C.g. vs. S.m. T1 0.092, T2 0.812 C.a. vs. S.m. T1 0.016 *, T2 0.571
		*C. glabrata*	72.7 (17.3)	94.3 (5.0)	0.232	
		*S. mutans*	33.1 (5.9)	95.7 (5.7)	0.008 *	

T1—laser settings (lower power); T2—laser settings (higher power); C.a.—Candida albicans; C.g.—Candida glabrata; S.m.—Streptococcus mutans; reduction in %CFU/mL—percentage reduction in the number of colony-forming units (CFU); SD—standard deviation; *—Statistical significance (*p* < 0.05)

**Table 7:** Percentage change (%) in the number of microbial cells (CFU/mL) after Nd:YAG laser application, compared to the control group (non-irradiated samples), for the C.g. + S.m., C.a. + S.m., and C.a. + C.g. planktonic mixtures.

Handpiece	Time	Bacteria	Reduction in %CFU/mLMean (SD)		*p*-Value T1 vs. T2	*p*-Value	*p*-Value DAI vs. 24AI
			T1	T2			
Genova C.g. + S.m.	DAI	*C. glabrata*	38.0 (10.2)	18.9 (0.8)	0.118	C.g. vs. S.m. T1 0.154, T2 0.002 *	*C. glabrata* T1 0.277, T2 0.001 * S. mutans T1 0.022 *, T2 0.001 *
	DAI	*S. mutans*	65.3 (13.8)	56.7 (2.3)	0.476		
	24AI	*C. glabrata*	14.3 (20.2)	0.0 (0.0)	0.423	C.g. vs. S.m. T1 0.423, T2 1.000	
	24AI	*S. mutans*	0.0 (0.0)	0.0 (0.0)	1.000		
Genova C.a. + S.m.	DAI	*C. albicans*	35.7 (2.5)	84.1 (2.2)	0.002 *	C.a. vs. S.m. T1 0.003 *, T2 0.513	*C. albicans* T1 0.003 *, T2 0.030 * S. mutans T1 1.000, T2 0.001 *
	DAI	*S. mutans*	0.0 (0.0)	81.8 (3.4)	0.001 *		
	24AI	*C. albicans*	0.0 (0.0)	12.5 (17.7)	1.000	C.a. vs. S.m. T1 1.000, T2 1.000	
	24AI	*S. mutans*	0.0 (0.0)	0.0 (0.0)	1.000		
Genova C.a. + C.g.	DAI	*C. albicans*	19.5 (7.4)	40.7 (0.9)	0.056	C.a. vs. C.g. T1 0.053, T2 0.081	*C. albicans* T1 0.157, T2 0.005 * C. glabrata T1 0.043 *, T2 0.038 *
	DAI	*C. glabrata*	46.8 (5.6)	54.0 (5.7)	0.326		
	24AI	*C. albicans*	33.4 (4.9)	18.2 (2.1)	0.056	C.a. vs. C.g. T1 0.086, T2 0.776	
	24AI	*C. glabrata*	7.4 (10.5)	20.0 (7.8)	0.305		

DAI—directly after irradiation; 24AI—24 hours after irradiation; T1—laser settings (lower power); T2—laser settings (higher power); C.a.—Candida albicans; C.g.—Candida glabrata; S.m.—Strepto-coccus mutans; reduction in %CFU/mL—percentage reduction in the number of colony-forming units (CFU)/mL; SD—standard deviation; *—Statistical significance (*p* < 0.05)

**Table 8:** Percentage change in the number of microbial cells (CFU/mL) after the application of the Nd:YAG laser to the C.g. + S.m., C.a. + S.m., and C.a. + C.g. two-species biofilms.

Handpiece	Time	Bacteria	Reduction in %CFU/mL Mean (SD)		*p*-Value T1 vs. T2	*p*-Value 24AI
			T1	T2		
Genova C.g. + S.m.	DAI	*C. glabrata*	69.5 (14.2)	99.9 (0.0)	0.094	*C. glabrata* vs. *S. mutans* T1 0.277, T2 1.000
	DAI	*S. mutans*	95.3 (1.3)	96.4 (4.9)	0.781	
Genova C.a. + S.m.	DAI	*C. albicans*	64.7 (22.9)	98.7 (0.3)	0.171	*C. albicans* vs. *S. mutans* T1 0.510, T2 0.031 *
	DAI	*S. mutans*	80.4 (15.8)	99.9 (0.1)	0.222	
Genova C.a. + C.g.	DAI	*C. albicans*	95.6 (3.2)	100.0 (0.0)	0.187	*C. albicans* vs. *C. glabrata* T1 0.032 *, T2 1.000
	DAI	*C. glabrata*	14.7 (20.8)	100.0 (0.0)	0.028 *	

DAI—directly after irradiation; T1—laser settings (lower power); T2—laser settings (higher power); C.a.—*Candida albicans*; C.g.—*Candida glabrata*; S.m.—*Streptococcus mutans*; reduction in %CFU/mL—percentage reduction in the number of colony-forming units (CFU)/mL; SD—standard deviation; *—Statistical significance (*p* < 0.05)

**Table 9:** Percentage (%) reduction in microbial cells (CFU/mL) after the application of the PBM parameters of the Nd:YAG laser (Genova handpiece) with the crystal violet method.

Handpiece	Bacteria	Reduction in %CFU/mL		*p*-Value
		T1 (SD)	T2 (SD)	
Genova(G)	*C. albicans*	12.2% (11.5)	33.5% (9.8)	0.184
	*C. glabrata*	30.4% (9.8)	34.6% (12.4)	0.741
	*S. mutans*	34.4% (1.8)	52.2% (1.0)	0.007 *
ANOVA: *p*-value		0.159	0.219	

T1—laser settings (lower power); T2—laser settings (higher power); reduction in %CFU/mL—percentage reduction in the number of colony-forming units (CFU)mL; *—Statistical significance (*p* < 0.05).

**Table 10:** Percentage (%) change in the number of microbial cells (CFU/mL) after the application of the Nd:YAG laser for the dual-species biofilm—crystal violet method.

Handpiece	Bacteria	Reduction in %CFU/mL		*p*-Value
		T1 (SD)	T2 (SD)	
Genova (G)	*C. albicans*	22.3% (7.9)	33.4% (0.1)	0.185
	*S. mutans*	18.4% (3.6)	31.6% (0.0)	0.035 *
ANOVA: *p*-value		0.585	0.001	
Genova (G)	*C. glabrata*	28.1% (5.7)	25.6% (0.0)	0.605
	*S. mutans*	17.3% (0.2)	22.1% (1.1)	0.028 *
ANOVA: *p*-value		0.117	0.049	
Genova (G)	*C. albicans*	28.2% (0.9)	55.3% (0.0)	0.001 *
	*C. glabrata*	21.5% (30.4)	41.3% (0.0)	0.455
ANOVA: *p*-value		0.784	<0.001	

T1—laser settings (lower power); T2—laser settings (higher power); reduction in %CFU/mL—percentage reduction in the number of colony-forming units (CFU)/mL; *—Statistical significance (*p* < 0.05).

## Data Availability

Data are contained within the article.

## References

[1] Li Y., Huang S., Du J., Wu M., Huang X. (2023). Current and prospective therapeutic strategies: Tackling Candida albicans and Streptococcus mutans cross-kingdom biofilm. Front. Cell Infect. Microbiol..

[2] Arweiler N.B., Netuschil L. (2016). The Oral Microbiota. Adv. Exp. Med. Biol..

[3] Zhang Y., Fang J., Yang J., Gao X., Dong L., Zheng X., Sun L., Xia B., Zhao N., Ma Z. (2022). Streptococcus mutans-associated bacteria in dental plaque of severe early childhood caries. J. Oral Microbiol..

[4] Brunke S., Hube B. (2013). Two unlike cousins: *Candida albicans* and *C. glabrata* infection strategies. Cell Microbiol..

[5] Lewis M.A.O., Williams D.W. (2017). Diagnosis and management of oral candidosis. Br. Dent. J..

[6] Bachtiar E.W., Bachtiar B.M. (2018). Relationship between Candida albicans and Streptococcus mutans in early childhood caries, evaluated by quantitative PCR. F1000Research.

[7] Lafuente-Ibáñez de Mendoza I., Cayero-Garay A., Quindós-Andrés G., Aguirre-Urizar J.M. (2021). A systematic review on the implication of Candida in peri-implantitis. Int. J. Implant Dent..

[8] Kanwar I., Sah A.K., Suresh P.K. (2017). Biofilm-mediated Antibiotic-resistant Oral Bacterial Infections: Mechanism and Combat Strategies. Curr. Pharm. Des..

[9] Baroni A., De Filippis A., Oliviero G., Fusco A., Perfetto B., Buommino E., Donnarumma G. (2018). Effect of 1064-nm Q-switched Nd:YAG laser on invasiveness and innate immune response in keratinocytes infected with *Candida albicans*. Lasers Med. Sci..

[10] Wiench R., Skaba D., Matys J., Grzech-Leśniak K. (2021). Efficacy of Toluidine Blue-Mediated Antimicrobial Photodynamic Therapy on Candida spp. A Systematic Review. Antibiotics.

[11] Grzech-Leśniak K. (2017). Making Use of Lasers in Periodontal Treatment: A New Gold Standard?. Photomed. Laser Surg..

[12] Grzech-Leśniak K., Matys J., Dominiak M. (2018). Comparison of the clinical and microbiological effects of antibiotic therapy in periodontal pockets following laser treatment: An in vivo study. Adv. Clin. Exp. Med..

[13] Grzech-Leśniak K., Matys J. (2021). The Effect of Er:YAG Lasers on the Reduction of Aerosol Formation for Dental Workers. Materials.

[14] Matys J., Grzech-Leśniak K., Flieger R., Dominiak M. (2016). Assessment of an Impact of a Diode Laser Mode with Wavelength of 980 nm on a Temperature Rise Measured by Means of k-02 Thermocouple: Preliminary Results. Dent. Med. Probl..

[15] Arnabat-Dominguez J., Vecchio A.D., Todea C., Grzech-Leśniak K., Vescovi P., Romeo U., Nammour S. (2021). Laser dentistry in daily practice during the COVID-19 pandemic: Benefits, risks and recommendations for safe treatments. Adv. Clin. Exp. Med..

[16] Golob Deeb J., Reddy N., Kitten T., Carrico C.K., Grzech-Leśniak K. (2023). Viability of bacteria associated with root caries after Nd:YAG laser application in combination with various antimicrobial agents: An in vitro study. Dent. Med. Probl..

[17] Nammour S., El Mobadder M., Maalouf E., Namour M., Namour A., Rey G., Matamba P., Matys J., Zeinoun T., Grzech-Leśniak K. (2021). Clinical Evaluation of Diode (980 nm) Laser-Assisted Nonsurgical Periodontal Pocket Therapy: A Randomized Comparative Clinical Trial and Bacteriological Study. Photobiomodul Photomed. Laser Surg..

[18] Grzech-Leśniak K., Matys J., Jurczyszyn K., Ziółkowski P., Dominiak M., Brugnera Junior A., Romeo U. (2018). Histological and Thermometric Examination of Soft Tissue De-Epithelialization Using Digitally Controlled Er:YAG Laser Handpiece: An Ex Vivo Study. Photomed. Laser Surg..

[19] Golob Deeb J., Smith J., Belvin B.R., Lewis J., Grzech-Leśniak K. (2019). Er:YAG Laser Irradiation Reduces Microbial Viability When Used in Combination with Irrigation with Sodium Hypochlorite, Chlorhexidine, and Hydrogen Peroxide. Microorganisms.

[20] Grönqvist A., Wiström J., Axner O., Monsen T.J. (2000). Bactericidal effect of pulsed 1,064 nm Nd:YAG laser light on Staphylococcus epidermidis is of photothermal origin: An in vitro study. Lasers Surg. Med..

[21] McCawley T.K., McCawley M.N., Rams T.E. (2022). Immediate effect of Nd:YAG laser monotherapy on subgingival periodontal pathogens: A pilot clinical study. J. Periodontal Implant. Sci..

[22] El Mobadder M., Nammour S., Namour M., Namour A., Grzech-Leśniak K. (2022). Disinfection Potential of 980 nm Diode Laser and Hydrogen Peroxide (3%) in “Critical Probing Depths” Periodontal Pockets: Retrospective Study. Life.

[23] Grzech-Leśniak K., Belvin B.R., Lewis J.P., Golob Deeb J. (2021). Treatment with Nd:YAG Laser Irradiation Combined with Sodium Hypochlorite or Hydrogen Peroxide Irrigation on Periodontal Pathogens: An In Vitro Study. Photobiomodul Photomed. Laser Surg..

[24] Yuanhong L., Zhongcheng L., Mengqi L., Daonan S., Shu Z., Shu M. (2016). Effects of Nd: YAG laser irradiation on the root surfaces and adhesion of Streptococcus mutans. Hua Xi Kou Qiang Yi Xue Za Zhi.

[25] Grzech-Leśniak K., Nowicka J., Pajączkowska M., Matys J., Szymonowicz M., Kuropka P., Rybak Z., Dobrzyński M., Dominiak M. (2019). Effects of Nd:YAG laser irradiation on the growth of Candida albicans and Streptococcus mutans: In vitro study. Lasers Med. Sci..

[26] Maden M., Görgül G., Sultan M.N., Akça G., Er O. (2013). Determination of the effect of Nd:YAG laser irradiation through dentinal tubules on several oral pathogens. Lasers Med. Sci..

[27] Kasić S., Knezović M., Beader N., Gabrić D., Malčić A.I., Baraba A. (2017). Efficacy of Three Different Lasers on Eradication of Enterococcus faecalis and Candida albicans Biofilms in Root Canal System. Photomed. Laser Surg..

[28] Krespi Y.P., Stoodley P., Hall-Stoodley L. (2008). Laser disruption of biofilm. Laryngoscope.

[29] de Paula Eduardo C., de Freitas P.M., Esteves-Oliveira M., Aranha A.C., Ramalho K.M., Simões A., Bello-Silva M.S., Tunér J. (2010). Laser phototherapy in the treatment of periodontal disease. A review. Lasers Med. Sci..

[30] Ge M.K., He W.L., Chen J., Wen C., Yin X., Hu Z.A., Liu Z.P., Zou S.J. (2015). Efficacy of low-level laser therapy for accelerating tooth movement during orthodontic treatment: A systematic review and meta-analysis. Lasers Med. Sci..

[31] Ren C., McGrath C., Yang Y. (2015). The effectiveness of low-level diode laser therapy on orthodontic pain management: A systematic review and meta-analysis. Lasers Med. Sci..

[32] Nakamura S., Okamoto M.R., Yamamoto K., Tsurumoto A., Yoshino Y., Iwabuchi H., Saito I., Maeda N., Nakagawa Y. (2017). The Candida species that are important for the development of atrophic glossitis in xerostomia patients. BMC Oral. Health.

[33] Jao Y., Ding S.J., Chen C.C. (2023). Antimicrobial photodynamic therapy for the treatment of oral infections: A systematic review. J. Dent. Sci..

[34] Namour M., Verspecht T., El Mobadder M., Teughels W., Peremans A., Nammour S., Rompen E. (2020). Q-Switch Nd:YAG Laser-Assisted Elimination of Multi-Species Biofilm on Titanium Surfaces. Materials.

[35] Risović D., Maver-Biśćanin M., Mravak-Stipetić M., Bukovski S., Bišćanin A. (2014). Quantitative investigation of efficiency of ultraviolet and visible light in eradication of Candida albicans in vitro. Photomed. Laser Surg..

[36] Rosa L.P., da Silva F.C., Viana M.S., Meira G.A. (2016). In vitro effectiveness of 455-nm blue LED to reduce the load of Staphylococcus aureus and Candida albicans biofilms in compact bone tissue. Lasers Med. Sci..

[37] Valm A.M. (2019). The Structure of Dental Plaque Microbial Communities in the Transition from Health to Dental Caries and Periodontal Disease. J. Mol. Biol..

